# Analyses of the Complete Genome and Gene Expression of Chloroplast of Sweet Potato [*Ipomoea batata*]

**DOI:** 10.1371/journal.pone.0124083

**Published:** 2015-04-15

**Authors:** Lang Yan, Xianjun Lai, Xuedan Li, Changhe Wei, Xuemei Tan, Yizheng Zhang

**Affiliations:** 1 College of Life Sciences, Sichuan University, Key Laboratory of Bio-resources and Eco-environment, Ministry of Education, Sichuan Key Laboratory of Molecular Biology and Biotechnology, Center for Functional Genomics and Bioinformatics, Chengdu, Sichuan, People's Republic of China; 2 Maize Research Institute of Sichuan Agriculture University, Key Laboratory of Crop Genetic Resource and Improvement, Ministry of Education, Wenjiang, Chengdu, People's Republic of China; Saint Mary's University, CANADA

## Abstract

Sweet potato [*Ipomoea batatas* (L.) Lam] ranks among the top seven most important food crops cultivated worldwide and is hexaploid plant (2n=6x=90) in the *Convolvulaceae* family with a genome size between 2,200 to 3,000 Mb. The genomic resources for this crop are deficient due to its complicated genetic structure. Here, we report the complete nucleotide sequence of the chloroplast (cp) genome of sweet potato, which is a circular molecule of 161,303 bp in the typical quadripartite structure with large (LSC) and small (SSC) single-copy regions separated by a pair of inverted repeats (IRs). The chloroplast DNA contains a total of 145 genes, including 94 protein-encoding genes of which there are 72 single-copy and 11 double-copy genes. The organization and structure of the chloroplast genome (gene content and order, IR expansion/contraction, random repeating sequences, structural rearrangement) of sweet potato were compared with those of *Ipomoea* (L.) species and some basal important angiosperms, respectively. Some boundary gene-flow and gene gain-and-loss events were identified at intra- and inter-species levels. In addition, by comparing with the transcriptome sequences of sweet potato, the RNA editing events and differential expressions of the chloroplast functional-genes were detected. Moreover, phylogenetic analysis was conducted based on 77 protein-coding genes from 33 taxa and the result may contribute to a better understanding of the evolution progress of the genus *Ipomoea* (L.), including phylogenetic relationships, intraspecific differentiation and interspecific introgression.

## Introduction

Chloroplasts (cp) are photosynthetic intracellular organelles and have their own genome of a circular double-stranded DNA molecule [1]. Since the first complete cp genome of liverwort (*Marchantia polymorpha*) was sequenced and characterized in 1986, more than 320 chloroplast genomes spanning 268 distinct organisms have been deposited in GOBASE (The Organelle Genome Database, http://gobase.bcm.umontreal.ca/) disclosing an enormous amount of cp evolutionary and functional information [2–4]. The first angiosperm cp genome with complete sequence was tobacco (*Nicotiana tabacum*), which has often been used as the reference genome in comparison to more recently studied angiosperm cp genomes [5]. Most cp genomes contain 110–130 genes encoding up to 80 unique proteins, about 30 tRNAs and 4 rRNAs. The majority of the protein-coding genes are related to photosynthesis as well as many biochemical processes in plant cells, such as the synthesis of sugar, lipid, amino acid, vitamin and pigment, starch storage, sulfate reduction and nitrogen metabolism [6]. It is also believed to have association with the plant's immune response [7]. The cp genome in angiosperms is usually 115–165 kb in size and has a quadripartite organization comprising of two 12–75 kb inverted repeats (IR) separating the 80–90 kb large single copy (LSC) and 16–27 kb small single copy (SSC) regions [8]. The cp genome contents and polycistronic transcription units are relatively conserved among the majority of the flowering plants. Since the substitution rate in cp-derived DNA is much lower than that in nuclear DNA in plants and the feature of uniparental inheritance, cp genomes are valuable sources for phylogenetic analysis of higher plants that are necessarily used for resolving complex evolutionary relationships [8,9]. However, along with the evolutionary process of the angiosperm species, cp genomes undergo recombination and rearrangements that resulted in deviations from the general rules. For example, the expansions and contractions of IR regions caused the variation in size among cp genomes [10]. In addition, structural and point mutations frequently occurred in cp genomes which are useful for comparative studies at intra- to inter-species levels [2]. In the previous comparative studies, gene loss-and-gain events and indels in the intergenic regions of cp genomes are identified in independent plant groups and have great potentials in addressing phylogenetic questions at both high and low taxonomic levels [7–11]. The evolutionary correlations between cp genome architecture and plant group diversification have been exemplified in a number of flowering plant groups, including *Fabaceae*, *Poaceae*, *Asteraceae*, *Gesneriaceae*, and *Oleaceae* [9,10]. Evolutionary hot spots, which are characterized by high incidences of indels and rearrangements, have been identified in different plant groups [11]. Therefore, cp genome sequences have been used to trace the evolutionary history of the plant kingdom, including phylogenetic relationships, intraspecific differentiation and interspecific introgression [2].

Sweet potato [*Ipomoea batatas* (L.) Lam] ranks among the top seven most important food crops cultivated worldwide due to its high and stable yield, rich nutrient content and strong stress resistance. It has the highest energy yields per unit area per unit time and more than 140 million tons are produced per year on about 9 million hectares in the world, over 97% of which are grown in developing countries. China accounts for 70% and 85% of the total area and yield of the world, respectively [12,13]. Sweet potato belongs to the family *Convolvulaceae*, genus *Ipomoea*, section *Batatas* and is the only hexaploid plant (2n = 6x = 90) in the *Convolvulaceae* family with a genome size between 2,200 to 3,000 Mb [14–16]. The genomic resources for this crop are deficient due to its complicated genetic structure. Even the evolutionary status of sweet potato and the phylogenetic relationships with other species in genus *Ipomoea* (L.) are not clear. An outstanding phylogeny question of the *Ipomoea* (L.) diversification is that the species like sweet potato with tuberous roots are found to scatter across the taxa of *Ipomoea* (L.), indicating the tuberous root characters have been independently derived multiple times. Many species unrelated to sweet potato have tuberous roots, but the sister clades of sweet potato in phylogenetic relationship are generally known to have fibrous roots, such as *I*. *purpurea* (L.) Roth, *I*. *nil* (L.) Roth, *I*. *pes-caprae* (L.) R.Br. Actually, on a fine scale, there are many closely related pairs of species in which one member has fibrous roots and the other produces tuberous roots such as *I*. *pubescens* (tuberous) and *I*. *purpurea* (fibrous), *I*. *plummerae* (tuberous) and *I*. *costellata* (fibrous) [17]. The evolutionary relationships among genus *Ipomoea* (L.) could be deduced using cp genome in phylogenetic analysis. In addition, the classic maternal inheritance of the cp genome makes the cytoplasmic markers potentially useful for analyzing the origin and evolution of the cultivated sweet potato, which might be spontaneous hexaploid intraspecific hybrids between diploid and tetraploid in our preliminary studies.

Here, we reported the complete cp genome sequence of sweet potato using the next-generation sequencing (NGS) technologies, as well as the assembly, annotation and the unique structure characterization of this cp genome. In addition, the organization and structure of sweet potato cp genome (gene content and order, IR expansion/contraction, random repeating sequences, structural rearrangement) were compared with those of *Ipomoea* (L.) species and some basal important angiosperms, respectively. Some boundary gene-flow and gene gain-and-loss events were identified from intra- to inter-specific levels. In addition, comparing with the transcriptome sequences of sweet potato, the RNA editing events and differential expression of the cp genes were detected. Moreover, we also performed phylogenetic analysis based on 77 protein-coding genes from 33 taxa which may contribute to a better understanding of the evolution progress of the genus *Ipomoea* (L.). It is sure that accessing to the genetic information of the sweet potato cp genome will not only improve the selection of germplasm process and the genetic large-scale breeding of this species, but also facilitate further usage of sequence data, such as phylogenetic and transplastomic analysis for the family of *Convolvulaceae*.

## Materials and Methods

### Plant materials, DNA extraction and sequencing

The sweet potato material used in this study was established from cultivar Xushu18 which is the leading cultivar in the south of China with long cultivated history. It has the thickening tuberous roots with red skin and white flesh and mainly used for starch accumulation and ethanol production [18]. Sweet potato is not considered a protected species and specific permissions were not required for collecting material in the specified location. Stem cuts of Xushu18 were planted in May, 2013 in experimental field of College of Life Sciences in Sichuan University, Chengdu, Sichuan Province of China.

Young leaves of plants were collected, snap-frozen immediately in nitrogen and stored at -80°C until further processing. The CTAB method was used for DNA extraction and purification [19]. For the Illumina HiSeq 2000 (GA II) platform, library construction and sequencing was provided at Beijing Genomics Institute (BGI)-Shenzhen, Shenzhen, China (http://www.genomics.cn). Sequencing libraries included paired-end library of 500 bp and mate-pair libraries with 2 kb insert sizes were constructed, resulting in 20G raw data of 90 bp sequenced reads. One lane of the flow cell was used for each sequencing library.

### *De novo* assembly and gap filling

The obtained nucleotide sequence reads were qualitatively assessed and assembled with *de novo* assemblers of Edena v2.1.1 (http://www.genomic.ch/edena.php) [20], SOAPdenovo2-r240 (http://soap.genomics.org.cn) [21] and Velvet v1.0.12 (http://www.ebi.ac.uk/~zerbino/velvet) [22] using different parameters, respectively. All of the assemblies from each assembler with optimized parameters were combined and treated with CD-HITEST to reduce redundancy (http://www.bioinformatics.org/cd-hit), and then the remains were reassembled with CAP3 (http://pbil.univ-lyon1.fr/cap3.php) [23]. Since the assembled contigs contain a mixture of sequences from both organellar and nuclear genomes, the methods as follows were used to isolate the chloroplast sequences based on the high correlation between contig read depth and the number of copies in the genome. Firstly, we sorted the assembled contigs by contig-read depth analysis of assemblies, that is, the raw reads sequence were mapped to the assembled contigs and the read depth of each contig was calculated through reads mapping. Taking the advantage of the difference of read depths among contigs, we could isolate the cp contigs with high-coverage (more than 2,000×) from the nuclear contigs. Then we confirmed those cp DNAs by BLASTing against *Ipomoea trifida* (Genebank: KF242496) cpDNA through Basic Local Alignment Search Tool (BLAST) using Blast2GO software v2.4.4 (http://www.blast2go.com/b2ghome) [24]. As result, 572 alternative contigs were hit to the reference cp genome in which the threshold was set to E-value≤10^-10^. In there, 8 contigs which overlapped with each other with average length at 30,290 bp consist of the circular whole cp genome of sweet potato. After the circular draft genome of the cpDNA was reconstructed, the Illumina reads were mapped to the draft genome to find sites of misassemblies and to correct the errors including homopolymers. Misassemblies can be detected as gaps or wrong-direction mate-paired reads. Therefore the draft genomes at the misassembled sites were broken and then repeated the contig-extension process described above. Mapping was performed using Bowtie2 program [25] available at the Galaxy website (http://main.g2.bx.psu.edu/) [26–28].

After all the errors and misassemblies corrected, the gaps between super-contigs of draft genomes were filled and verified by PCR amplification and Sanger sequencing. The primers for amplification (S1 Table) were designed according to the contig sequences or homologous sequence alignments using Primer Premier 5.0 (PREMIER Biosoft International, CA, USA) and synthesized by GENEWIZ, Inc. (http://www.genewiz.com.cn). The gap genes were amplified using KOD FX DNA polymerase (TOYOBO, Japan) and the PCR products were fractionated and recovered in a 1% agarose gel, then ligated to 50ng vector pMD-18T (TIANGEN BIOTECH, Beijing, China) using T4 DNA ligase (TAKARA BIO, Japan). Recombinant plasmids were transformed into Escherichia coli JM109 competent cells and clones were picked for validation through colony PCR, plasmid electrophoresis and restriction enzyme digestion (Fermentas, USA). The positive plasmids were sequenced at BGI-Shenzhen, Shenzhen, China. The completed chloroplast sequence was deposited in GenBank with the accession number in KP212149.

### Genome annotation and analysis

Preliminarily gene annotation was carried out through the online program Dual Organellar GenoMe Annotator (DOGMA) with plastid/bacterial genetic code and default conditions [29]. To verify the exact gene and exon boundaries, MUSCLE [30] was used to align putative gene sequences with their homologues acquired from BLAST searches in GenBank. All tRNA genes were further confirmed through online tRNAscan-SE and tRNADB-CE search server [31–33]. The graphical map of the circular plastome was drawn with Organellar Genome DRAW (OGDRAW v1.2) [34]. In the analysis of sweet potato cp genome, the REPuter program was used to visualize direct and inverted repeats under the criteria cutoff n≥17 bp, 90% sequence identities, and non-overlapping regions [35]. The frequency of codon usage was calculated from exon sequences of all protein-coding genes in the sweet potato cp genome. In the comparison of *Ipomoea* (L.) plants cp genome, 19 species were whole-genome aligned using MultiPipMaker [36]. The sweet potato cp genome was compared with cp genomes of *Arabidopsis thaliana*, *Glycine max*, *Ipomoea trifida*, *Nicotiana tabacum*, *Oryza sativa*, *Populus trichocarpa*, *Ricinus communis*, *Solanum tuberosum*, *Sorghum bicolor*, *Vitis vinifera and Zea mays* using a Mauve software [37].

### Phylogenetic analysis

Phylogenetic analysis was performed on the basis of 77 chloroplast genes from 33 species (chloroplast genome accession were in S2 Table). Alignments were performed by MAFFT version 5.0 with default parameters after all positions containing gaps or missing data being eliminated. The statistical method of Maximum Likelihood (ML) and the computer program phyML version 3.0 were applied for phylogenetic reconstruction, with parameters estimated from the data [38]. The general time reversible (GTR) substitution model was selected for ML analysis, taking in account the gamma distribution of rate heterogeneity with four discrete categories [39]. Branch support was evaluated by 1,000 replications of bootstrap (BS) re-sampling.

### RNA extraction for transcriptome analyses

The trancriptome information for RNA editing and differential expression analysis were obtained from the transcriptome database of vegetative organs of cultivar XS 18 built by our laboratory. Total RNAs were extracted using the Trizol Reagent (Invitrogen, USA), and treated with DNase I (Fermentas, USA) according to the manufacturer’s instructions. RNA quality and purity were assessed with OD_230/260_ ratio. Total cDNAs were synthesized from RNA with Moloney murine leukemia virus (M-MLV) reverse transcriptase (Invitrogen, CA, USA) using oligo (dT) as primer following the manufacturer’s instructions. Two kinds of RNA sample were submitted to Illumina GA II platform for sequencing at Beijing Genomics Institute (BGI)-Shenzhen, Shenzhen, China (http://www.genomics.cn). The raw reads were assembled using Trinity release_2013-08-24 with default parameters.

### RNA editing and differential expression analyses

The cDNA reads were mapped to the protein-coding genes of the cp genome using bowtie2 software and transcriptome sequences were aligned against the assembled cp genome using local Blast program. The cDNA sequences of cp genes were in comparison with the DNA sequences for the RNA editing events. The annotation nomenclature of RNA editing sites is according to the principle in PREPACT [40]. Some RNA editing sites were verified by reverse transcription polymerase chain reaction (RT-PCR) experiments and sequenced using Sanger sequencing. Primer sequences were given in S1 Table.

For the differential expression analyses of cp genes in XS 18 vegetative organ, we first mapped the reads in transcriptome databases of leaves and stems to the assembled transcripts of the vegetative organs database. The calculating of cp genes expression was using the util/alignReads.pl script in Trinity software [41]. In order to compare the expression abundance among samples, units were normalized to FPKM (fragments per kilo bases per million reads). To get deeper statistics of the expression among young leaves (YL), mature leaves (ML) and stems, we used the digital gene expression (DGE) tag profiling which had been built by our laboratory in 2012 [42]. According to all the tags generated from three sequenced DGE libraries of sweet potato, we searched CATG with the downstream 17 base pairs in the assembled cp transcripts and the resulted 21 base pairs tags became the new expression tags related to the cp genes. These tags were mapped to the distinct clean tags in DGE tag profiling of transcriptome and the resulted cp gene expression tags were aligned to the cp gene sequences using Bowtie available at the Galaxy website to detect the expression level of cp genes. In order to compare the expression abundance among samples, tags were normalized to TPM (number of transcripts per million clean tags).

The edgeR package (Empirical analysis of Digital Gene Expression in R) was used for the above two differential expression analysis of cp genes [43]. We normalized tag distribution for gene expression level in each library to make an effective library size and extracted differentially expressed transcripts (DETs) with p value≦0.05 and log2 fold-change≧1. And we compared libraries pair-wise and used hypergeometric test to identify DETs.

### Validation of the differentially expressed transcripts

Real-time PCR-based relative quantification was used to validate the DETs in differential expression analyses. The SYBR Green based real-time PCR primer sets were designed using Beacon Designer 3.0 (Premier Biosoft International, CA) and listed in S1 Table. Primers stocks were prepared at 100 μM in TE (10mM Tris, pH 8.0, 1mM EDTA), and working solutions were diluted to 10 μM. RNA extraction and reverse transcription of YL, ML and stem were performed as the methods above. The gene beta-actin was used as reference to normalize the amount of total RNA present in each reaction. When using the ΔΔCt method to calculate relative gene expression, it is necessary for the same amplification efficiency between target and reference genes [44]. Dilutions were generated from target and reference gene sample diluted 10-fold in order to calculate individual efficiency (E) based on slope of the line (E = 10^-1^/slope) considering an ideal value range of 0.90 to 1.05 [44–46].

All real-time PCR runs were performed in three technical replicates and each reaction mixture was prepared using SYBR Premix Ex Taq kit (Takara). PCR amplifications were carried out in a total volume of 20μl, containing 6.4 μl PCR-grade water, 0.8 μl of each primer, 10 μl 2×SYBR Premix Ex Taq, and 2.0 μl appropriately diluted template cDNA. The thermal cycling protocol was as follows: initial denaturation for 10 min at 95°C followed by 40 cycles of 5 s at 95°C, 5 s at 55°C, and 5 s at 72°C. The fluorescence signal was measured at the end of each extension step at 72°C. After the amplification, a melting peak analysis with a temperature gradient of 0.1°C per second from 60 to 95°C was performed to confirm that only the specific products were amplified. These procedures were optimized for 96-well format using a Bio-Rad IQ detection system which using fluorescein as an internal passive reference dye for normalization of well-to-well optical variation.

## Result

### Organization and gene content of sweet potato cp genome

**Fig 1:**
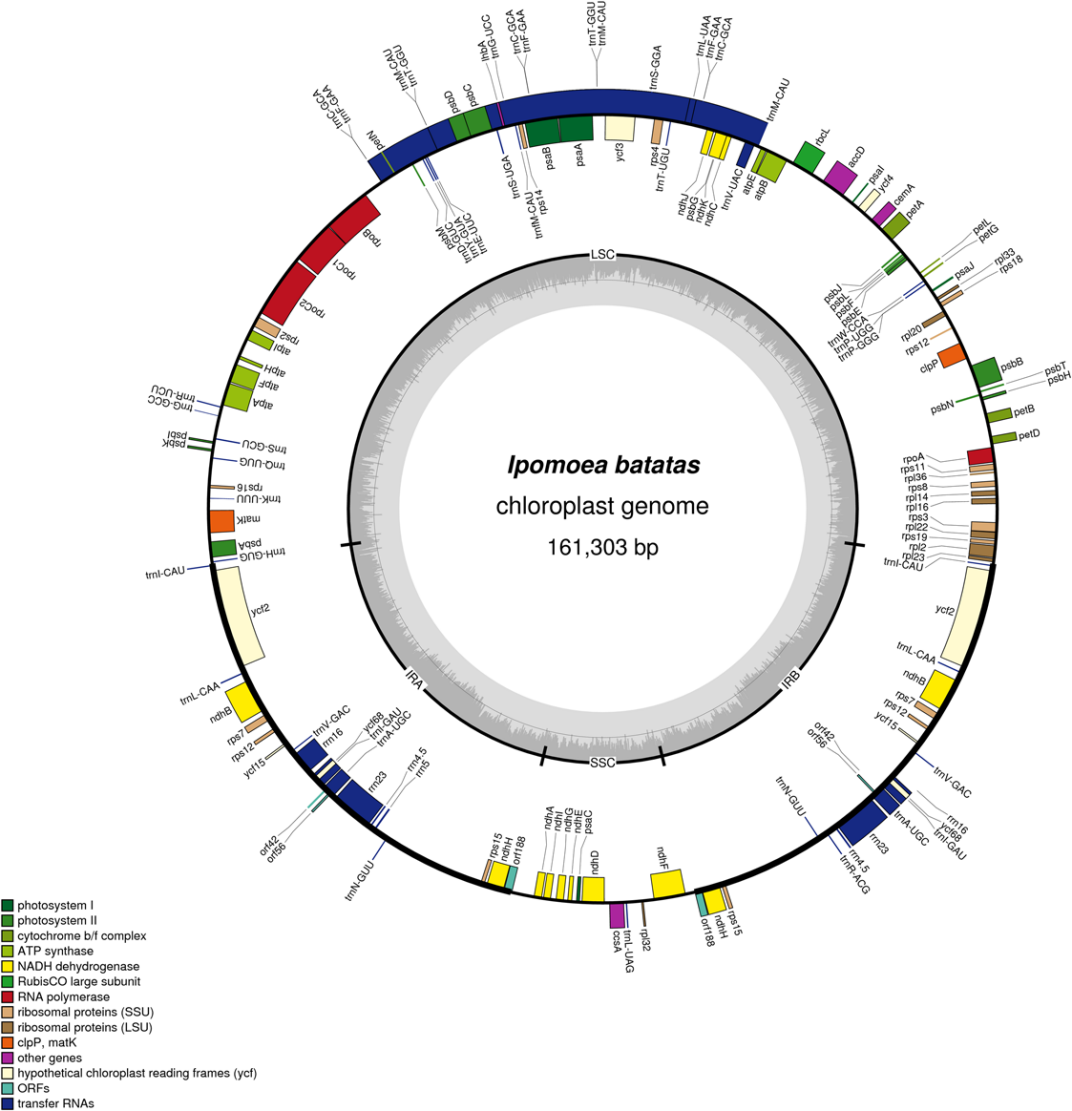
Chloroplast genome of *Ipomoea batats*. The outer circle shows positions of genes in the large single copy (LSC), small single copy (SSC), and two inverted repeat (IR A and IR B) regions. The inner circle is a graph depicting GC content across the genome. Plastome maps were generated in OGDraw v1.2.

The cp genome of sweet potato is a circular molecule of 161,303 bp and is structured in the typical quadripartite structure, consisting of two inverted repeats (IRA and IRB) separated by large (LSC) and small single copy (SSC) regions (Fig 1). The GC content of the cp DNA is 38.45%, similar to the other reported cp genomes from the family of *Convolvulaceae*. The GC content of the LSC and SSC are 36.13% and 33.78%, respectively, whereas that of the IR regions is 41.25%. The sweet potato cp genome contains a total of 145 genes among which 103 have one single copy and 21 are duplicated. Table 1 shows the survey of genes in the cp genome. Apart from 8 duplicated rRNA genes (rrn23, rrn16, rrn5 and rrn4.5) and 43 tRNA genes (31 single-copy and 6 two-copy genes), there are 94 protein-coding genes among which there are 72 single-copy genes located in LSC/SSC regions and 11 two-copy genes in IRs. These protein-coding genes are approximately classified into 5 groups. The first group has 36 genes related to photosynthesis, including photosystem I and II, cytochrome b6/f complex, ATP synthase, Calvin clycle and C-type cytochrome related genes. The second group contains all chloro-respiration-related genes for the synthesis of the NADH-dehydrogenase complex. The 11 *ndh* genes include 9 single-copy genes within LSC region (*ndhC*, *K* and *J*) or SSC region (*ndhF*, *D*, *E*, *G*, *I*, *A*) and 2 two-copy genes located within the IRs (*ndhB*, *H*). The third group has 26 genes related to the gene expression machinery involved in transcription, splicing and translation, including maturase K, RNA polymerase, ribosomal large and small subunit related genes. The forth group has 3 genes with single-copy for metabolic pathway regulation (*accD*, *clpP*, *cemA*). Finally, there are 7 pseudogenes with unknown function in which *ihbA* is a unique gene relative to other *Ipomoea* (L.) plants.

**Table 1:** Functional genes encoded by the sweet potato cp genome (72 single-copy genes and 11 two-copy genes).

Groups	Functional System	Num	Gene names
Photosynthesis	Photosystem I	7	*psaA* ^[^ ^b^ ^]^, *psaB*, *psaC*, *psaI*, *psaJ*, *ycf3* ^[^ ^c^ ^]^, *ycf4*
	Photosystem II	15	*psbA*, *psbB*, *psbC*, *psbD*, *psbE*, *psbF*, *psbG*, *psbH*, *psbI*, *psbJ*, *psbK*, *psbL*, *psbM*, *psbN*, *psbT* ^[^ ^b^ ^]^
	Cytochromeb6/f complex	6	*petA*, *petB*, *petD*, *petG*, *petL*, *petN*
	ATP synthase	6	*atpA*, *atpB*, *atpE*, *atpF* ^[^ ^b^ ^]^, *atpH*, *atpI*
	Calvin clycle	1	*rbcL*
	C-type cytochrome synthesis	1	*ccsA*
Chloro-respiration	NADH oxidoreductase	11	*ndhA* ^[^ ^b^ ^]^, *ndhB* ^[^ ^a^ ^,^ ^b^ ^]^, *ndhC*, *ndhD*, *ndhE*, *ndhF*, *ndhG*, *ndhH* ^[^ ^a^ ^]^, *ndhI*, *ndhJ*, *ndhK*
Expression machinery	RNA polymerase	4	*rpoA*, *rpoB*, *rpoC1* ^[^ ^b^ ^]^, *rpoC2*
	ribosomal large subunit	9	*rpl14*, *rpl16*, *rpl2*, *rpl20*, *rpl22*, *rpl23*, *rpl32*, *rpl33*, *rpl36*
	ribosomal small subunit	12	*rps2*, *rps3*, *rps4*, *rps7* ^[^ ^a^ ^]^, *rps8*, *rps11*, *rps12* ^[^ ^a^ ^,^ ^b^ ^,^ ^d^ ^]^, *rps14*, *rps15* ^[^ ^a^ ^]^, *rps16*, *rps18*, *rps19*
	maturase K	1	*matK*
metabolic pathway	acetyl-CoA carboxylase carboxyltransferase	1	*accD*
	clp protease proteolytic subunit	1	*clpP*
	chloroplast envelope membrane protein	1	*cemA*
pseudogenes	unknown functions	7	*ycf2* ^[^ ^a^ ^]^, *ycf15* ^[^ ^a^ ^]^, *ycf68* ^[^ ^a^ ^]^, *lhbA*, *orf42* ^[^ ^a^ ^]^, *orf56* ^[^ ^a^ ^,^ ^b^ ^]^, *orf188* ^[^ ^a^ ^]^

Superscript

^[a]^ means the gene in the IR region with two copies;

^[b]^ means the gene contains a single intron;

^[c]^ means the gene contains two introns;

^[d]^ means the gene divided into two independent transcription units.

In addition, the cp genome has 11 genes harbouring introns among which 8 are located in the LSC (6 protein-coding and 2 tRNA genes) and the rest 3 are in the IRs. The number of introns in sweet potato cp genome is lower than that in other Angiosperms plants (e.g. 18 genes with introns in *Elaeis guineensis* [1] and 15 genes in *Arbutus unedo* [47]). In these 11 genes, there are 10 with one single intron (8 protein-coding and 2 tRNA genes), whereas *ycf3* contains two introns. Similarly, the cp DNA of sweet potato contains a lower number of codons (21,713) in comparison with other angiosperms (e.g. *Ageratina adenophora* with 24,894 [48] and *Vigna radiata* with 26,274 [49]). This is possibly due to the pseudogenization of multiple ORFs in the sweet potato cp genome and the loss of the 6 kb *ycf1* which is the same as the Poaceae species but much different from other *Ipomoea* (L.) plants [17,47]. Table 2 shows the frequency of codon usage deduced on the protein-coding gene sequences. It is demonstrated that leucine is the most frequent amino acid with 2,330 codons, while cysteine is the least frequent with 280 codons. The codon usage in sweet potato displays the obviously preference as it is biased toward high representation of A and T at the third codon position, as observed in most land plant cp genes.

**Table 2:** Codon usage of the sweet potato cp genome.

Amino acid	Codon	Number	Amino acid	Codon	Number
Ala	GCA/GCU/GCC/GCG	343/412/449/125	Met	AUG	526
Cys	UGU/UGC	192/88	Asn	AAU/AAC	749/190
Asp	GAU/GAC	598/276	Pro	CCA/CCU/CCG/CCC	329/358/252/56
Glu	GAA/GAG	842/283	Gln	CAA/CAG	537/242
Phe	UUU/UUC	939/342	Arg	CGA/CGU/CGG/CGC/AGA/AGG	312/334/87/68/408/140
Gly	GGA/GGU/GGC/GGG	538/589/312/126	Leu	UUG/UUA/CUA/CUU/CUC/CUG	405/694/476/382/270/103
His	CAU/CAC	407/131	Ser	UCA/UCU/UCG/UCC/AGU/AGC	681/477/309/187/302/70
Ile	AUA/AUU/AUC	546/1078/185	Val	GUU/GUA/GUC	543/412/286
Lys	AAA/AAG	798/243	Trp	UGG	380
Thr	ACA/ACU/ACG/ACC	551/379/163/56	Tyr	UAA/UAC	529/238

### Cp genome restructure among genus *Ipomoea* (L.)

**Fig 2:**
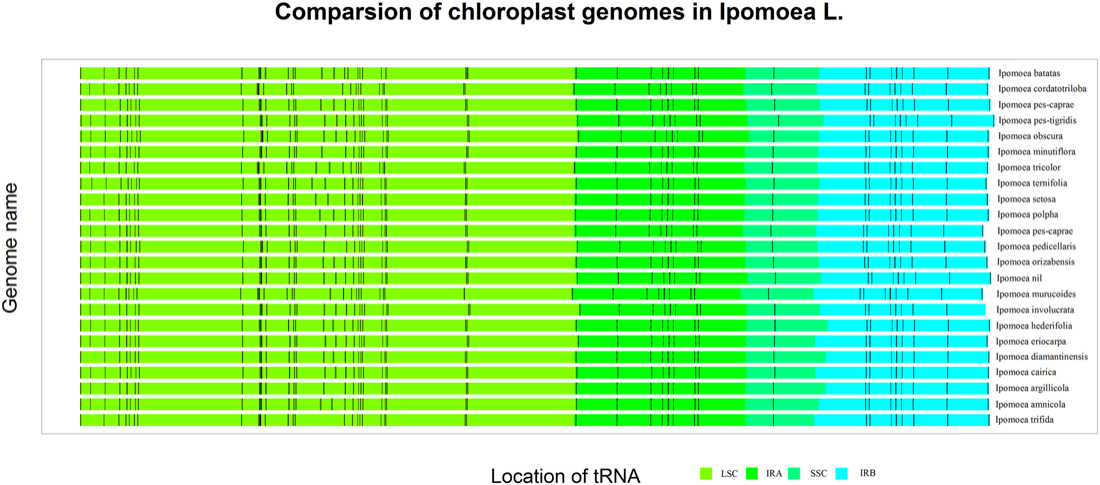
Chloroplast genome alignment among species in *Ipomoea* genus. The relative position of tRNA genes were determined and marked as short vertical line in black. The thickness of the vertical bar represented the relative length of the tRNA genes. The LSC, SSC and IRs regions of each chloroplast genome were shown in four different colors.

**Fig 3:**
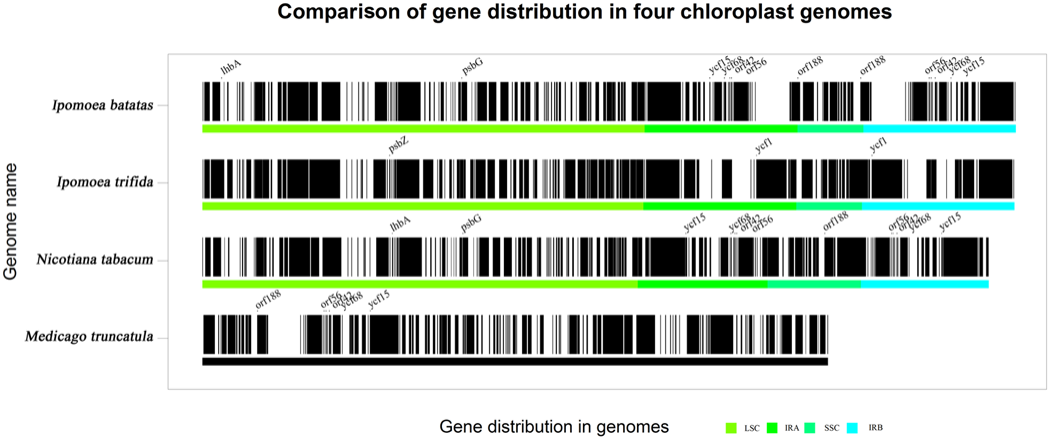
Comparison of gene distribution in chloroplast genomes of sweet potato and other three inter- and intra-*Ipomoea* species. The relative position of functional-protein encoding genes were determined and marked as short vertical line in black. The thickness of the vertical bar represented the relative length of the genes. The name and position of some differential genes between two genomes were marked. The LSC, SSC and IRs regions of each chloroplast genome were shown in four different colors (except for *Medicago truncatula* with incomplete genome).

*Ipomoea* (L.) is an emerging model system represented by *I*.*trifida* and *I*.*nil* [17]. Resolving the phylogenetic relationships of genus *Ipomoea* (L.) is critical for understanding their evolution. The whole-genome alignment of sweet potato cp genome with that of other *Ipomoea* (L.) plants (Fig 2) showed high conservation in coding regions along with remarkable rearrangements of tRNA gene orders and positions. Although sweet potato is extremely close to the *I*.*trifida* in evolutionary relationship, the cp DNA of sweet potato clearly deviates from that of *I*.*trifida* because of extensive rearrangements. The length of sweet potato cp genome is larger than that of *I*.*trifida* with about extra 300 bp resulted from the 7 additional tRNA genes in the LSC region, which are responsible for transporting leucine, valine, arginine and proline. In addition, two novel protein-coding genes, *lhbA* and *psbG*, are detected in the LSC region and some ORFs like *orf42*, *orf56* and function-unknown protein-coding genes are found in the IRs of sweet potato cp genome (Fig 3). In addition, the length comparisons of the different regions within *Ipomoea* (L.) plants revealed a remarkable shortness of the SSC region in *I*.*trifida* due to the shortness of the entire *ndhA* operon crossing SSR and IR regions.

Another remarkable rearrangement in sweet potato cp genome is the *ycf* genes, which encode unknown-functional proteins, contain introns and are regarded as pseudogenes. Similar to *I*.*trifida*, the sweet potato cp genome also contains the *ycf2* within the IR regions. However, it loses the two-copy *ycf1* comparing with *I*.*trifida* and other *Ipomoea* (L.) plants (Fig 3). Since the *ycf1* or *ycf2* presents a trend of deterioration in some higher plants like *Elaeis guineensis* and *Arbutus unedo* [1,47], the loss of *ycf1* in sweet potato could be seen as a sign of evolution in cultivated species. In addition, the functions of hypothetical *ycf15* and *ycf68* are ambiguous in various land plants. To prove these two genes exist commonly in plants, a multiple alignments of the cp genome were conducted among sweet potato, *Nicotiana tabacum* and *Medicago truncatula* (Fig 3). The result showed that the conserved region of *ycf15* and *ycf68* in sweet potato could be read through without internal stop codon. Due to the lack of the start codon, these two genes formed uncompleted ORFs encoding peptides of 63 and 108 amino acid residues, respectively. However, these two genes vary a lot in different species. For example, *Ipomoea purpurea* and *Ageratina adenophora* had completed *ycf15* ORF encoding RF15 protein, but the former had no *ycf68* while the latter had one uncompleted *ycf68* ORFs as sweet potato. In *Musa acuminata*, these two genes were not functional since there were several stop codons in different positions (S1 Fig). The annotation against the NCBI-Nr database of these two genes in sweet potato displayed that *ycf15* is similar to NADH-quinone oxidoreductase of *Medicago truncatula*, whereas *ycf68* is similar to *ycf133* in many plants including *Oryza sativa* and *Zea mays*. It is worth noting that there are only sweet potato and *I*.*purpurea* having *ycf15* and *ycf68* in genus *Ipomoea* (L.), demonstrating some common points in their functions and evolutionary processes.

The result from the repeat structure analysis using REPuter showed that hundreds of random repeated sequences were presented in sweet potato cp genome. As the ubiquitous and unstable genomic elements, tandem repeats could be used as a genetic marker to distinguish two species. In sweet potato cp genome, there are a mass of random repeats in which some large motifs are widespread (117–213 nt). The largest one is a 236 nt forward repeat sequence located in intergenic spacer (IGS). Except for the multiple IGS-repeat sequence, there were 13 directed, inverted and palindromic random repeats in protein-coding sequences. These random repeats ranged from 17 to 39 nt in size among which 7 were located in functional protein-coding region, 2 in intronic regions and 4 in tRNA genes. The identifications of the seven random repeats in coding region are important because mutations in these repeats often have phenotypic consequences [47]. Locations and directions of these 13 random repeat sequences were listed in Table 3. In the mean while, random repeats in sweet potato, *I*.*nil* and *I*.*trifida* could be used in the genetic research. There were 8 repeats found at the same locations in these three species, while two pairs of directed repeats located at AccD-AccD and AtpI- IGS (trnN-GUU-rps15) are only present in sweet potato which could be used as unique genetic markers.

**Table 3:** Repeat sequences and their distribution in cp genome of sweet potato.

Repeat type	Repeat size	Repeat1 position	Repeat2 position	Repeat1 location	Repeat2 location	Repeat sequence
Forward	39	45230–45269	99641–99680	Intron (*ycf3*)	IGS (*rps12*-*ycf15*)	CAGAACCGTACATGAGATTTTCACCTCATACGGCTCCTC
	38	59760–59798	59784–59822	*AccD*	*AccD*	GAAAGTTCTAATGAGAATGAAAGCGAAAGTTCTAATGA
	36	40125–40161	42350–42386	*PsaB*	*PsaA*	AATAGCTAAATGATGGTGTGCAATATCGGTCAGCCA
	22	9654–9676	37813–37835	trnG-GCC	trnG-UCC	GATGCGGGTTCGATTCCCGCTA
	22	8124–8146	36869–36891	trnS-GCU	trnS-UGA	AGAGAGGGATTCGAACCCTCGG
	20	54022–54042	144734–144754	trnV-UAC	trnA-UGC	GCTCTACCAACTGAGCTATA
	20	15073–15093	113223–113243	*AtpI*	IGS (trnN-GUU-*rps15*)	AAAGAAACAAGAACAACAA
Reverse	26	6039–6065	14796–14822	IGS(*rps16*- trnQ-UUG)	*atpH*	TCATGAATAGTCATAGGTTCTATTAT
	25	1614–1639	37917–37942	IGS (*psbA*- *matK*)	*PsaB*	AAATAAAAAAAAAAAAAAGAATTCT
	17	76295–76312	127586–127603	*PsbT*	*NdhG*	CGGCGAGAAATCTTTAT
Palindromic	39	45230–45269	149439–149478	Intron (*ycf3*)	IGS (*ycf15*- *rps12*)	CCAGAACCGTACATGAGATTTTCACCTCATACGGCTCCT
	28	8120–8148	46916–46944	trnS-GCU	trnS-GGA	TGGAAAGAGAGGGATTCGAACCCTCGGT
	19	15073–15092	135861–135880	*AtpI*	IGS(*rps15*- trnN-GUU)	TAAAGAAACAAGAACAACA

IGS: intergenic spacer

### Boundary gene-flow and structural comparison within angiosperms

**Fig 4:**
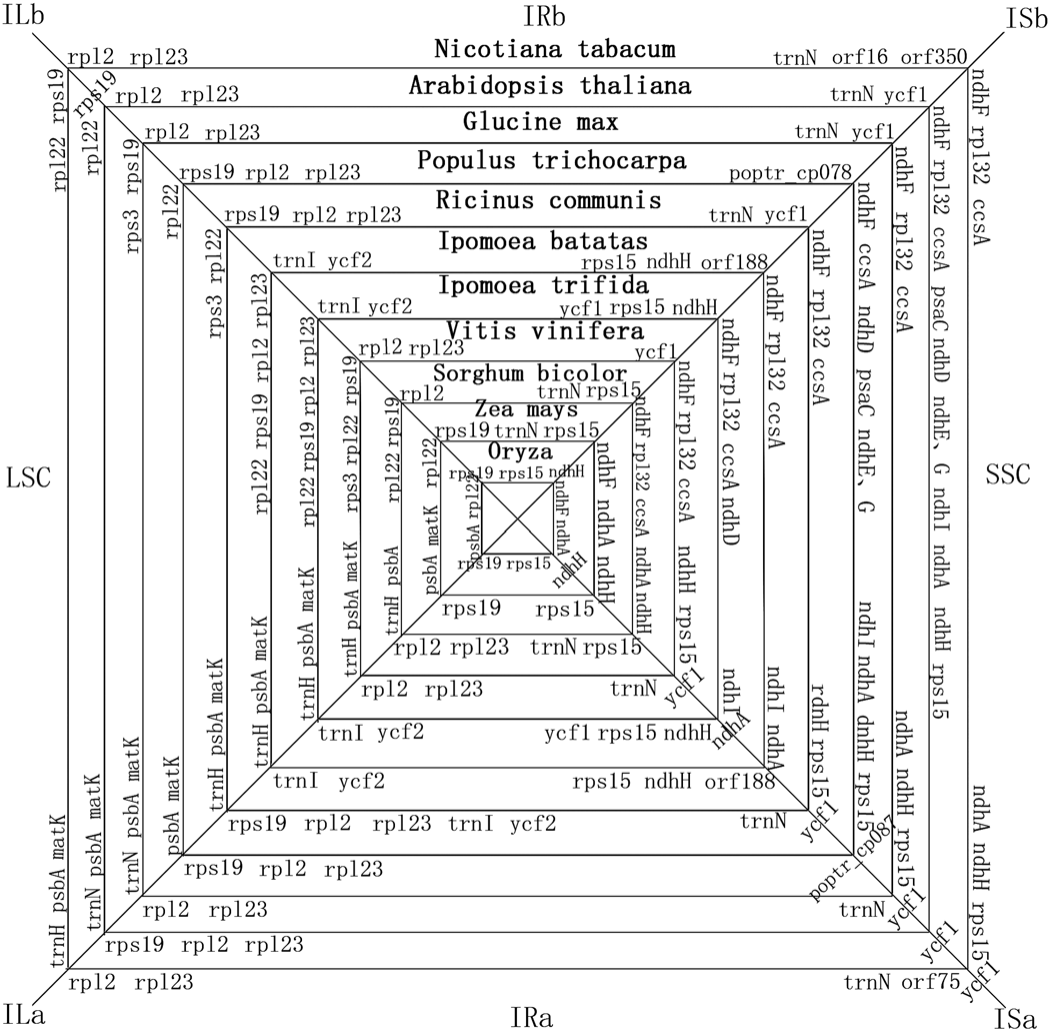
Boundary gene-flow and IR region expansion/contraction events. Comparison of the junction positions of IR boundaries among 11 basal angiosperms cp genome. ILa, ILb represented the positions between the two IRs and the LSC region, ISa and ISb represented the positions between the two IRs and the SSC region.

The gene content and order in the cp genomes of most angiosperms are generally conserved. However, as the availability of more sequenced genomes, a number of IR region expansion-and-contraction events have been identified. Although IRs are the most conserved regions and the rate of neutral nucleotide substitutions in IRs is lower than that in single-copy regions, variation between IR/LSC and IR/SSC boundaries is the main reason for size variation among cp genomes of different taxa. The two IRs in cp genomes have four junctions, ILa, ILb (between the two IRs and the LSC region), ISa and ISb (between the two IRs and the SSC region). The events of gene flow and single-double gene transfer occurred in these four boundaries of 11 different angiosperm cp genomes (eight dicotyledons and three monocotyledons) were shown in Fig 4. The most remarkable boundary gene-flow event was found in ILb where *rps19* flowed from IRB to LSC among these species. As shown in Fig 4, *rps19* in *Populous trichocarpa* and *Ricinus comminis* are located downstream of the ILb boundary, being the first gene in IRB region followed by *rpl2* and *rpl23*. However, *rps19* flowed across the ILb boundary in *Arabidopsis thaliana* and became totally single-copy gene in LSC region in *Nicotiana tabacum*, *Glycine max* and *Vitis vinifera*. This IRs contraction is very apparent in Convolvulaceae presented by *Ipomoea batats* and *Ipomoea trifida* since *rps19* along with *rpl2* and *rpl23* flowed into LSC region together. Similarly, in monocotyledons, *Oryza sativa* and *Zea mays* harbored the duplication of *rps19* in IRB but it flowed into LSC in *Sorghum bicolor*. Besides contraction, IRs expansion occurred in ISb boundary in which *rps15* and *ndhH* flowed from SSC to IRB region. In most dicotyledons, *rps15* and *ndhH* were in SSC closed to IRA region while these two single-copy genes were found in duplication in IRB region of Convolvulaceae species. Therefore, in *I*.*batatas* and *I*.*trifida*, the contraction of IRB region caused by *rps19*, *rpl2* and *rpl23* were repaired in length by the joining of *rps15* and *ndhH*. However, in monocotyledons like *O*.*sativa*, *Z*.*mays* and *S*.*bicolor*, *rps15* was in IRB region of these three species and *ndhH* duplicated only in *O*.*sativa*. It demonstrated that the species with most double-copy genes in IR regions was *O*.*sativa* and those with the less IR-genes were *N*.*tabacum*, *G*.*max* and *V*.*vinifera*. Although the evolutionary relationship is close, *I*.*batatas* and *I*.*trifida* have subtle difference in gene order and IR variation. For example, *ndhA* gene was located totally in SSC of *I*.*batatas* while flowed across to IRA in *I*.*trifida*.

### Gene gain-loss events and phylogenetic analysis

**Fig 5:**
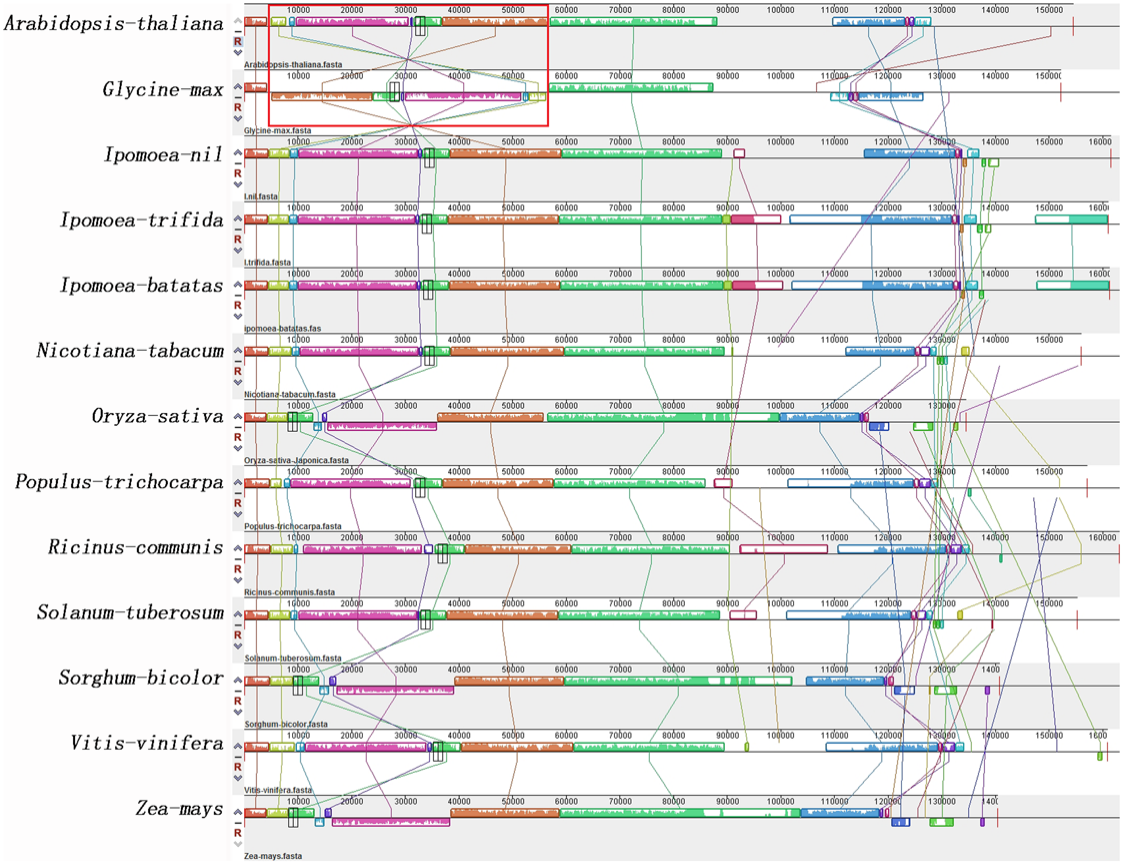
Gene map and genome alignment of 13 basal important angiosperms species. MAUVE multiple alignment implemented in Geneious. Colored outlined blocks surround regions of the genome sequence that aligned with part of another genome. The coloured bars inside the blocks are related to the level of sequence similarities. Lines link blocks with homology between two genomes.

The whole cp genome alignment between sweet potato and other angiosperms showed high conservation in many coding regions along with remarkable exceptional gene gain-loss events. Many gene-losses have been interpreted as transferring to the nucleus. For example, the following genes were lost in different species: the *rpl32* in the *Populus* genus, *rps16* in *Medicago truncatula*, *rpl33* in *Phaseolus vulgaris*, *ycf1*, *ycf2* and *accD* in Poaceae [47]. Fig 5 showed the co-localization of cpDNAs on chromosomes of 13 different species (shared or conserved synteny). The gene orders in these cp genomes were compared using *N*.*tabacum* as reference because it is considered to have the ancestral angiosperm gene order. As shown in Fig 5, the cpDNA of sweet potato clearly deviated from that of *N*.*tabacum* because of extensive expansion and rearrangements. The higher divergence is observed in the region of 90,000 to 110,000 bp as well as the terminal (145,000–160,000 bp), including the IR and SSC regions. Besides, the gene number and order around 130,000 bp varies a lot between *N*.*tabacum* and *Convolvulaceae* plants. In the region before 90,000 bp, the majority of dicotyledons exhibited a conserved synteny within the LSC regions but *G*.*max* showed extensive rearrangements, resulting in a considerable loss of synteny. Monocotyledons represented by *O*.*sativa*, *Z*.*mays* and *S*.*bicolor* exhibited their unique conserved synteny which is different from dicotyledons. From the alignment results, it is concluded that the LSC region of the monocotyledons cp genomes had one segment inversions (10,000–30,000 bp) and *G*.*max* had at least five inversions.

**Fig 6:**
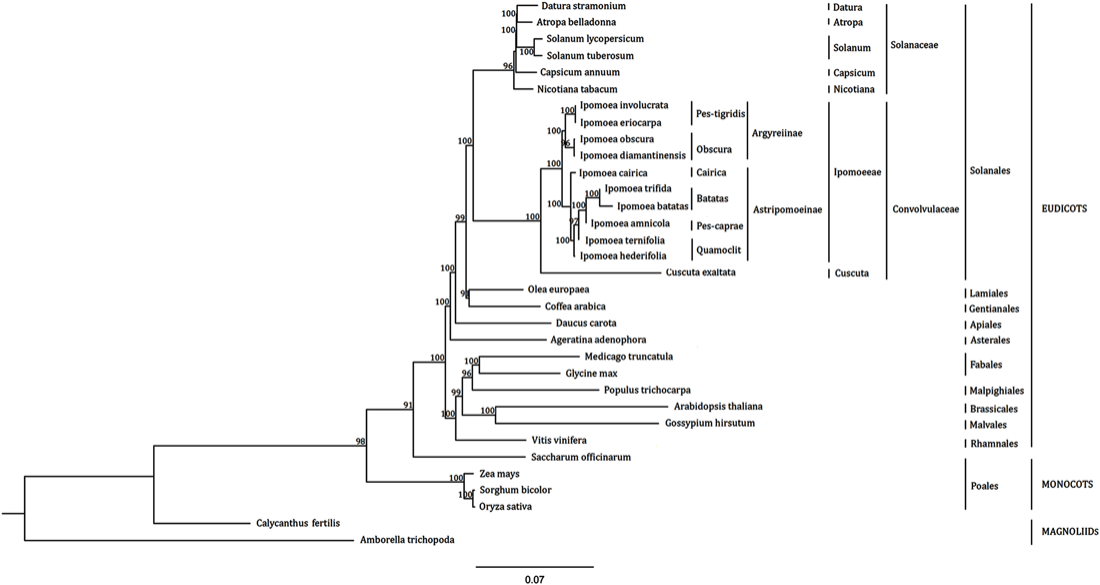
Phylogram based on sequence analysis of 77 chloroplast genes from 33 plant species. Numbers above each node indicate the ML bootstrap support values. The current taxonomic classifications are indicated on the right.

The phylogenetic reconstruction was built upon 77 protein-coding genes of 33 angiosperms cp genomes, supported by three major monophyletic groups: magnoliids, monocots, and eudicots (Fig 6). These 77 genes in each of the 33 species were with average 94,359 nucleotides and 84,089 after gap removal and the problem with missing data from the sequence alignment was minimized. The resulting phylogenetic topologies of the maximum likelihood analysis were with high bootstrap supports and provided the evolutionary placement and relationship of sweet potato in angiosperms clades. Within eudicots, ten *Ipomoeeae* species had very close relationship but diverged from other *Convolvulaceae* species like Cuscuta (*Cuscuta exaltata*). It is demonstrated that *I*.*batats* and *I*.*trifida* are sister clades and have the closest phylogenetic relationship inside the Ipomoeeae. Besides *Convolvulaceae* species, multiple edible industrial crops like potato (*Solanum tuberosum*), tomato (*Solanum lycopersicum*) and chili (*Capsicum annuum)* reperenting *Solanales* were analysed. *Solanales* had closer relationship with *Lamiales* (*Olea europea*), *Gentianales* (*Coffea arabica*), *Apiales* (*Daucus carota*) and *Asterales* (*Ageratina adenophora*) in *Asterids* than other *Rosids* species like *Fabales* (*Glycine max*), *Malpighiales* (*Populus trichocarpa*), *Malvales* (*Gossypium hirsutum*) and *Brassicales* (*Arabidopsis thaliana*). As regards with monocots, four *poales* species had relatively close phylogenetic relationship in which topologies are strongly supported with 100% bootstrap values in the ML trees. In spite of that, the relationship between *Sorghum bicolor* and *Oryza sativa* were closer than that with *Zea mays*, whereas *Saccharum officinarum* was somewhat alienated and classified into the other clade. In addition, the phylogenetic relationship of two Magnoliids cp genomes (*Calycanthus fertilis* and *Amborella trichopoda*) strongly supported that Monocots and Eudicots are sister clades with Magnoliids diverging before the Monocots-Eudicots split. Therefore, our results are congruent with the previous phylogenetic analyses among genera of Angiosperms.

### RNA editing events searched by comparison with transcriptome sequences

Taking use of the transcriptome sequences of sweet potato built by our laboratory in 2012 [42], we compared the cp protein-coding sequences with 128,052 transcripts to identify the RNA editing events in sweet potato cp genome. The cp protein-coding sequences matched 398 out of 128,052 transcripts (0.31%), which is equivalent to 5× coverage of the cp coding region. Totally 43 RNA editing sites were found in 27 protein-coding genes including 40 in exons and 3 in introns with only one site in *ycf3*, *petB* and *rps16*, respectively. The highest number of RNA editing events was found in *ycf2* and *rps3* with 5 followed by *ndhB* with 4. In addition, the majority of editing events were at the second site of codons followed by the third and the first one, which is consistent with codon position bias in plant RNA editing. All the RNA editing sites were listed in Table 4.

**Table 4:** Position of RNA editing sites in chloroplast transcripts of sweet potato.

Gene	Cp genome positon	Gene position	AA position	Triplet position	Bases	Codon amino	acid change	Label
*rps16*	5007	976	326	1	C→U	CUA→UUA	L→L	rps16eU976LL
*rps16*	5755	226	76	-	C→U	UGC→UGU	-	rps16iU226CC
*psbK*	7440	136	46	1	C→U	CUC→UUC	S→L	psbKeU136SL
*atpA*	11006	914	305	2	C→U	UCA→UUA	S→L	atpAeU914SL
*atpF*	13143	92	31	2	C→U	CCA→CUA	P→L	atpFeU92PL
*rps2*	16508	134	45	2	C→U	ACA→AUA	T→I	rps2eU134TI
*rpoB*	26998	338	113	2	C→U	UCU→UUU	S→F	rpoBeU338SF
*psbD*	34240	53	18	2	C→U	ACU→AUU	T→I	psbDeU53TI
*rps14*	38487	149	50	2	C→U	CCA→CUA	P→L	rps14eU149PL
*ycf3*	44366	1668	556	-	C→U	GAC→GAU	-	ycf3iU1168DD
*ndhJ*	50916	320	107	2	C→U	GCG→GUG	A→V	ndhJeU320AV
*ndhK*	51549	683	228	2	U→C	AUG→ACG	M→T	ndhKeC683MT
*ndhK*	51601	697	233	1	C→U	CUU→UUU	L→F	ndhKeU697LF
*ndhK*	52001	231	77	3	U→C	AAU→AAC	N→N	ndhKeC231NN
*ndhC*	52196	278	93	2	C→U	GCA→GUA	A→V	ndhCeU278AV
*accD*	59371	232	78	1	C→U	CAU→UAU	H→Y	accDeU232HY
*accD*	59681	551	184	2	C→U	ACU→AUU	T→I	accDeU551TI
*cemA*	63358	242	81	2	C→U	CCA→CUA	P→L	cemAeU242PL
*petA*	64560	527	176	2	C→U	GCU→GUU	A→V	petAeU527AV
*psbE*	66431	214	72	1	C→U	CCU→UCU	P→S	psbEeU214PS
*rpl33*	69762	20	7	2	C→U	GCA→GUA	A→V	rpl33eU20AV
*rps18*	70166	33	11	3	C→U	UCC→UCU	S→S	rps18eU33SS
*rpl20*	70956	131	44	2	C→U	GCU→GUU	A→V	rpl20eU131AV
*clpP*	73106	324	108	3	C→U	GCC→GCU	A→A	clpPeU324AA
*petB*	77516	296	98	-	C→U	UCA→UUA	-	petBiU296SL
*petB*	78586	611	204	2	C→U	CCA→CUA	P→L	petBeU611PL
*rps3*	85153	609	203	3	C→U	AUC→AUU	I→I	rps3eU609II
*rps3*	85423	339	113	3	U→C	GUU→GUC	V→V	rps3eC339VV
*rps3*	85486	276	92	3	C→U	ACC→ACU	T→T	rps3eU276TT
*rps3*	85520	242	81	2	C→U	CCA→CUA	P→L	rps3eU242PL
*rps3*	85710	52	18	1	C→U	CAU→UAU	H→Y	rps3eU52HY
*rpl23*	87527	194	65	2	C→U	ACA→AUA	T→I	rpl23eU194TI
*ycf2*	88475	243	81	1	U→C	UUU→CUU	F→L	ycf2eC243FL
*ycf2*	89627	1395	465	3	G→A	UCG→UCA	S→S	ycf2eA1395SS
*ycf2*	89907	1676	559	2	U→C	UUA→UCA	L→S	ycf2eC1676LS
*ycf2*	90243	2011	671	1	G→A	GAA→AAA	E→K	ycf2eA2011EK
*ycf2*	91075	2843	948	2	C→U	UCC→UUC	S→F	ycf2eU2843SF
*ndhB*	95864	1481	494	2	C→U	CCA→CUA	P→L	ndhBeU1481PL
*ndhB*	96509	830	277	2	C→U	UCA→UUA	S→L	ndhBeU830SL
*ndhB*	97273	737	246	2	C→U	CCA→CUA	P→L	ndhBeU737PL
*ndhB*	97861	149	50	2	C→U	UCA→UUA	S→L	ndhBeU149SL
*rps12*	99558	144	48	3	C→U	GCC→GCU	A→A	rps12eU144AA
*ndhH*	117572	726	242	3	C→U	UAC→UAU	Y→Y	ndhHeU726YY

In the 43 RNA editing sites in sweet potato, there were 36 C-to-U changes, which was the most common editing sites, followed by 5 U-to-C and 2 G-to-A. As reported before, C-to-U change is the most predominant form in chloroplasts and mitochondria of seed plants and the reverse U-to-C editing is rarely observed in seed plants [3]. Interestingly, the U-to-C editing sites were found twice in *ycf2* and *ndhK* transcripts and once in *rps3* of sweet potato. As to the controversial G-to-A editing, both 2 editing sites were found in *ycf2* transcript. The G-to-A change in the *ycf2* reported few in cp genome of higher land plants before and some scholars consider it the sequencing error which may overestimate the number of RNA editing events [1]. However, with the increasing number of cp genome sequencing and G-to-A change found in more plants like *E*.*guineensis* and *A*.*thaliana* [1,8], G-to-A editing events in sweet potato should not be accidental events.

The consequence of RNA editing may result in the alteration of the amino acid sequence. There were 31 non-synonymous substitutions in 43 editing events, which led to the protein alteration. The most frequent non-synonymous substitutions were found in the *ycf2* and *ndhB* transcript with 4 editing sites. Apart from the *ycf2* transcript with unknown function, ndhB transcripts were found to be highly edited in other plants such as *N*.*tabacum* and *A*.*thaliana*. Comparing the editing site of *ndhB* transcripts among *N*.*tabacum*, *A*.*thaliana* and *I*.*batats*, the highly edited ndhB transcripts maintained the conserved amino acid sequences, indicating that the RNA editing process is limited and conserved among higher plant species.

### Differential expression of sweet potato cp genes

Two transcriptome databases including leaves and stems of sweet potato were established using RNA-Sequencing to explore the expressions of cp genes in different tissues. The expression levels of total 83 cp protein-coding genes were calculated by mapping all the reads of each transciptome database to the identified cp genome. FPKM (fragments per kilo bases per million reads) was used as the standardized unit of the gene length and sequencing depth to make the expression levels of genes in different transcriptomes comparable. From the overall situation of expression, there were 76 genes expressed in one or two tissues while 7 not expressed. Moreover, the expression level varied a lot in these expressed genes as there were 24 genes expressed highly with >100 FPKM in average while 10 had extremely low level (with <1 FPKM in average). Interestingly, the highest expressed genes like *rbcL* (2614.54 and 439.33 FPKM in leaves and stems respectively), *psbD* (1172.21 and 307.62 FPKM) and *petG* (952.73 and 288.86 FPKM) were photosynthesis-related genes, followed by chloro-respiration-related genes like *ndhB* (599.6 and 196.55 FPKM). The other three kinds of genes had relatively lower expression, especially in rps-like genes which encode the ribosomal small subunits related to gene expression machinery, the average expression was lower than 1 FPKM. For example, *rps19* had the expression with 1 and 0.82 FPKM in leaves and stems respectively, *rps15* with 0.3 and 0.06 FPKM and *rps11* with 0.91 and 0.09 FPKM. However, the whole expression levels of ribosomal large subunits rpl-like genes were not the same. Except for *rpl22* which was expressed lowly with 0.16 FPKM in average, the rpl series had relatively higher expression such as *rpl20* with 25.52 FPKM and *rpl32* with 23.99 in average.

**Fig 7:**
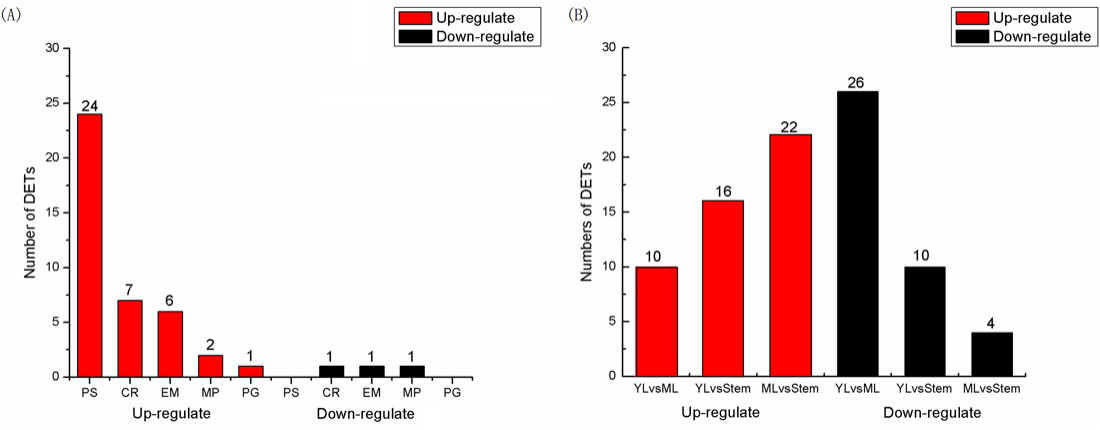
Differentially expressed transcripts (DETs) of cp genes in sweet potato. (A) Numbers of DETs in leaves and stems of sweet potato. PS, CR, EM, MP, PG represented genes related to photosynthesis, chloro-respiration, expression machinery, metabolic pathway and pseudogenes. (B) Numbers of DETs among young leaves (YL), mature leaves (ML) and stems in sweet potato. The Up-(red) and down-regulated (black) statistics of DETs were calculated in edgeR.

In order to explore the differential expression of cp functional genes among tissues, the differential expression analyses were performed using edgeR. There were 43 out of 83 functional genes displaying different expression levels between leaves and stems, which were named differential expression transcripts (DTEs) [42]. Fig 7A shows the DTEs in five functional types as photosynthesis (PS), chloro-respiration (CR), expression machinery (EM), metabolic pathway (MP) and pseudogenes (PG). It is worth noting that the majority of DTEs were up-regulated in leaves and even rose to a degree that some genes expressed more than 10 times in leaves than that in stems. For example, *atpB* gene expressed highly with 60.93 FPKM in leaves while lowly with 4.04 FPKM in stems. In addition, the PS-DETs were the foremost differential expression type which accounted for 60% (24/40) of all the up-regulated DTEs and 71% (24/34) of all the PS-genes in cp genome. Unlike other types of DETs having down-regulating members in leaves, all of the PS-DETs were up-regulated, indicating that the PS-genes in leaves indeed had higher expression than in stems due to the stronger ability of photosynthesis. As verifying and supplement, the digital gene expression (DGE) tag profiling was also used to analyze the expression difference in three vegetative tissues as young leaves (YL), mature leaves (ML) and stems. From the overall perspective, the highly expressed genes also focus on the photosynthesis- and chloro-respiration-related genes like *rbcL* (37.43 TPM, tags per million reads), *psbD* (31.97 TPM), *psbA* (26.22 TPM) and *ndhF* (28.05 TPM). Oppositely, the extremely-low expressed genes like *rps16* (0.08 TPM), *rpoB* (0.29 TPM) and *rps7* (0.27 TPM) were related to the RNA polymerase and ribosomal subunit. In addition, we found a number of differentially-expressed cp genes among the three tissues, especially between the young and mature leaves. There were totally 34 out of 83 genes differentially expressed. For example, *ndhF* gene expressed highly in YL (73.38 TPM) while relatively low in ML (8.46 TPM) and *rbcL* gene with 7.75 TPM in YL while 50.96 TPM in ML. To analyze differential expression patterns of cp genes between each two tissues, including the up-regulated and down-regulated genes, we pair-wisely compared them and obtained 3 pairs of comparisons (Fig 7B). The results showed that there were 10 genes up-regulated while 26 down-regulated in YL in the YLvsML, and 22 genes up-regulated and only 4 down-regulated in ML in the MLvsStem.

## Discussion

### *De novo* assembly of the complete chloroplast genome of sweet potato

Since chloroplasts are maternally inherited and have unique features for reconstructing the phylogenetic relationships among organisms, cp genomes have been broadly used in phylogenetic studies [50]. Fortunately, the recent progress in next-generation sequencer (NGS) technology makes the whole-genome sequencing easily performed. Here, using NGS data generated by Illumina HiSeq 2000, we performed the de novo assembly and annotation of sweet potato cp genome. Unlike the traditional cp genome sequencing method, the genomic DNA was extracted from fresh green leaves of adult plants, sequenced and assembled to contigs. The cp genome sequences were then achieved by super-contigs assembling from the contig database using the known reference cp genome. Moreover, deep coverage from NGS data could distinguish the chloroplast sequences from the nucleus sequences and the misassembled contigs. We sorted the assembled contigs by depth of the coverage and confirmed the high-coverage contigs (more than 2,000×) to be the cp DNAs by BLASTing against *I*.*trifida* cpDNA. As reported before, assembled sequences could be classified into cpDNA, mtDNA and nuclear genomes on the basis of the coverage depth: 2,000–3,000× for cpDNA, ~200× for mtDNA and ~20× for nucleus [20,50]. This step ensured more accurate classification and precise location of cp genome than directly using raw reads to distinguish cp genome.

In order to ensure the integrity and accuracy of re-assembled super-contigs, the Illumina reads were mapped onto the draft genome to find sites of misassemblies and to correct the errors including homopolymers. Since there is no perfect assembler program so far, de novo assembly processes always generate misassembled contigs which can be detected as gaps or wrong-direction mate-paired reads, sometimes it could be unexpected insert sizes [50]. The super-contigs at the misassembled sites were broken and then repeated the contig-extension process described above. The assembled contigs must be double-checked by read-mapping and be scanned for any gaps of lower coverage or of unexpected directions/distances of paired-reads. The manually-closed gaps between super-contigs were filled and verified using PCR followed by Sanger-sequencing further. Therefore, we have determined the complete cp genome of sweet potato using this assemble-and-mapping process.

### Cp genomes resolve evolutionary relationships in angiosperms and identify genome-scale evolutionary patterns in phylogenetic analyses

Angiosperms are the largest clades of land plants with >250,000 species distributed in nearly every terrestrial habitat [51]. Many phylogenetic studies have been based on DNA sequences of individual genes before and remain either incompletely resolved or weakly supported. We performed phylogenetic analyses of 77 cp genes in 33 sequenced cp genomes to estimate relationships among the major angiosperm clades and the resulting trees are used to examine the evolution at cp genome level. The former researches had plenitudinous evidence through model-based approaches for the position of *Amborella* as the earliest diverging lineage of flowering plants, followed by *Nymphaeales* and *Austrobaileyales* [51]. They also provided strong support for parallel relationship between eudicots and monocots, and *Austrobaileyales* to be the next-diverging lineage which is sister to a clade containing Chloranthales and magnoliids [51,52]. The phylogenetic analysis through cp genome sequences confirmed the above diversification relationship by a more reliable method, for cpDNAs are much more suitable for phylogenetic studies in a broad range of species.

As for the diversification relationship and evolutionary status of sweet potato in *Convolvulaceae*, this study mainly focused on the monophyletic tribe Ipomoeeae which consists of ca. 650–900 species distributed throughout the tropics and subtropics of the world [17]. Phylogenetic result provided the evolutionary relationships among *Ipomoeeae* lineages, which strongly supported for *I*. *batatas* and *I*. *trifida* as sister to one another. Although these lineages have no obvious distinguishing morphological features, their difference in fibrous and tuberous root characters was the biggest contradiction. It supported previous findings that *Ipomoeeae* lineages morphology is evolutionarily labile. The characteristics of the tuberous roots are found to scatter across the taxa of *Ipomoea* [17]. From our understanding of phylogenetic relationships for these species, we can deduce that tuberous roots have been independently derived in multiple times.

It's worth noting that sweet potato was always phylogenetic closed to *S*. *tuberosum*, *P*.*trichocarpa* and *G*.*max* in evolutionary analysis of various individual genes. They had been considered to be the allied species since the identity and coverage of the protein sequence in some genes were more than 90% among these species. However, in cp genome level, these species belonged to different genera, indicating that they did not have closely phylogenetic relationship actually. The reason for the high clustering in previous phylogenetic trees could be either the conversation of the protein-coding sequence itself or lack of much closer candidate sequences for the phylogenetic analyses. Therefore, the evolutionary relationship should be studied at genome level with population genes instead of individual genes.

### The evolutionary roles of cp RNA editing events in the divergence of plants

In land plants, the RNA editing events are known to take place in the transcripts of chloroplasts. Before translation, coding messages of cp genomes can be interfered through RNA splicing and editing [40]. As our result, the most common edition in sweet potato is the cytidine to uridine (C to U) type of nucleotide exchange at the second site of codons. The majority of RNA editing sites affects codon identities resulting in non-synonymous substitutions. Moreover, it is possible that there has been a loss of conserved editing sites among species. For example, potato had nine C-to-U conversions, five of which resulted in amino acid changes while tomato had seven C-to-U conversions with all resulted in an amino acid change [53]. What evolutionary or functional significance might be attributed to these findings? With the increasing cp genome sequence data, it revealed the striking similarity in the conservative nature with regard to the same quadripartite structure and a similar set of gene content. As reported before, in the comparison of different cp genome sequences, promoters, intergenic regions and replication origins are well conserved, as are the coding regions [54]. In contrast, RNA editotypes differ between the two species both qualitatively and quantitatively. RNA editing may a rapidly evolving trait with editing sites changing quickly between taxa. It thus fulfils all requirements for a major trait in speciation because it may have a substantial effect on plant fitness via protein function. The species- and site-specific RNA editing events which provide chloroplast markers are indispensable in species evolutionary process. Relying on this kind of posttranscriptional processing, plants could regulate their cp gene expressions that cause incompatibility of genetic compartments among species to generate the divergence in the process of plant speciation [54].

### Differential expression revealed the functional labor division among cp genes

**Fig 8:**
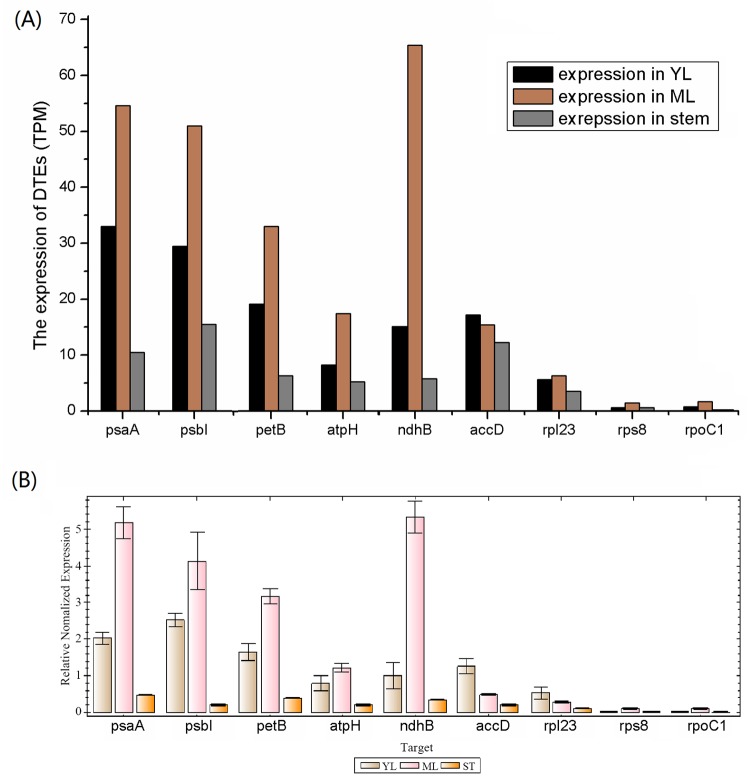
Expression levels of some DETs in young leaves (YL), mature leaves (ML) and stems. (A) The expression of 9 DETs identified in digital gene expression profile. (B) Validation the expression of these DETs in relative real-time PCR-based quantification.

The analysis of gene differential expression is an important method to understand their functional division in the process of evolution or hybridization. In this study, photosynthesis- and chloro-respiration-related genes had higher expression level than the other functional genes, which affirmed that photosynthesis is the prominent role of chloroplast. Moreover, the stronger photosynthesis organ showed much higher expression of same genes. This was confirmed by the fact that some genes were expressed generally higher in ML than in YL and stem, and that numerous genes expressed in ML were up-regulated and the specifically-expressed genes were almost found in ML. Fig 8 showed the expression levels of some representative DETs in YL, ML and stem. All these nine DETs were validated through real-time quantitative PCR.

Indeed, almost all of the photosynthesis-related genes stabilized at a higher expression level than the others. It heralds that these genes were co-regulated by the same mechanism. Actually, many of the genes located in the cp genome are arranged within polycistronic transcription units and transcribed under the control of one promoter [55,56]. The functional significance of the gene clustering in polycistronic units is not understood but this organization may facilitate the stoichiometric production of related subunits. For example, PSII subunits encoding genes are located within five operons, *psbA*, *psbB*-*psbT*-*psbH*-*petB*-*petD*, *psbE*-*F*-*L*-*J*, *psbD*-*psbC* and *psbI*-*psbK*. The *psbB* operon contains genes for the PSII (*psbB*, *psbT*, *psbH*) and cytochrome b6f (*petB* and *petD*) complexes [56]. Since this operon has a single prokaryotic-like promoter in the upstream of the *psbB*, the expression of these genes were stable with 105.88, 114.97, 92.26, 129.63 and 126.76 FPKM, respectively.

*RbcL*, one of the photosynthesis-related genes, had the highest expression in leaves among all the cp-genes with 2614.51 FPKM. This gene encodes the large subunit of ribulose-1,5-bisphosphate carboxylase in higher plants and has a strong core promoter itself which is sufficient to obtain wild-type rates of transcription [57]. Moreover, the transcription of photosystem I and II genes is mediated by a plastid-encoded RNA polymerase (PEP). The accumulation of PEP transcripts at a high level from poorly conserved promoters depends on upstream activators such as the light-responsive promoter of the *psbD* gene encoding the D2 photosystem II subunit protein [55–58]. Such a positive feedback makes photosystem I and II genes expressed highly as the expression of *psbD* with 1172.21 FPKM in leaves. Although the photosynthesis-related genes expressed highly in leaves, their expression level in stem had significant difference (*rbcL* and *psbD* genes with 439.33 and 430.75 FPKM in stems). It has reported that the transcription rates of *rbcL* gene were up to 10-fold higher in light-grown leaves than in dark-adapted stems [57,59]. The high transcription rate of photosynthesis-related genes in leaves might be related to the σ^70^-Type promoters of PEP which is light regulated in chloroplasts [59].

However, except for the positive feedback mechanism which regulates the PEP expression, the control of cp gene expression includes several processes that are similar to those of prokaryotic and/or eukaryotic systems. PEP itself is encoded by *rpoA*, *rpoB*, *rpoC1* and *C2* genes which was lowly expressed in sweet potato with 4.22 FPKM in average [55]. That means that the PEP accumulation may not be enough through its expression and regulation. In some reports before, there is a second nuclear-encoded transcription activity in chloroplasts (NEP, nuclear-encoded plastid RNA polymerase) supplementary to PEP [60]. NEP is a phage-type monomeric RNA polymerase which preferentially transcribes housekeeping genes in general. However, in some species like *C*. *gronovii* and *C*. *subinclusa* which have lost the *rpo* genes coding for the PEP subunits, a number of adaptations within the plastid genome were required to enable gene transcription mediated exclusively by the NEP [57]. So that NEP has to be also responsible for the expression of photosynthesis-related genes at levels sufficient to allow for photosynthesis. The regulation mechanism of PEP positive feedback and the NEP substitution through interaction or complement is not ascertained yet.

## Conclusion

We assembled, annotated and analyzed the complete chloroplast sequence of sweet potato (*Ipomoea batatas*). This cp genome was compared to other available *Ipomoea* (L.) cp genomes and some basal important angiosperms. The comparison showed that the orders and contents of cp genes were conserved except some structural rearrangements during evolutionary process identified through some boundary gene-flow and gene gain-and-loss events. The identification of repetitive sequences and RNA editing sites which could be used as new chloroplast markers within *Ipomoea* species opens new perspective to refine the phylogeny of *Ipomoea* (L.) and the origin of cultivated hexaploid sweet potato. Taken together, this study provides new insight in the evolutionary relationships inter- and intra- morning glory species and established a foundation for future studies.

## Supporting Information

S1 Fig The ORF alignments of *ycf15* and *ycf68* genes in different cp genomes. (TIF)

S1 Table Lists of primers used to complete gap regions, RT-PCR and qRT-PCR. (DOC)

S2 Table Accessions for taxa used in phylogenetic reconstruction and genome comparison in this study. (DOC)
