# Supplementary figures and images for: Analyses of the Complete Genome and Gene Expression of Chloroplast of Sweet Potato [Ipomoea batata]
Source: PLoS One. 2015 Apr 15;10(4):e0124083. doi: 10.1371/journal.pone.0124083 (PMC4398329; doi:10.1371/journal.pone.0124083)

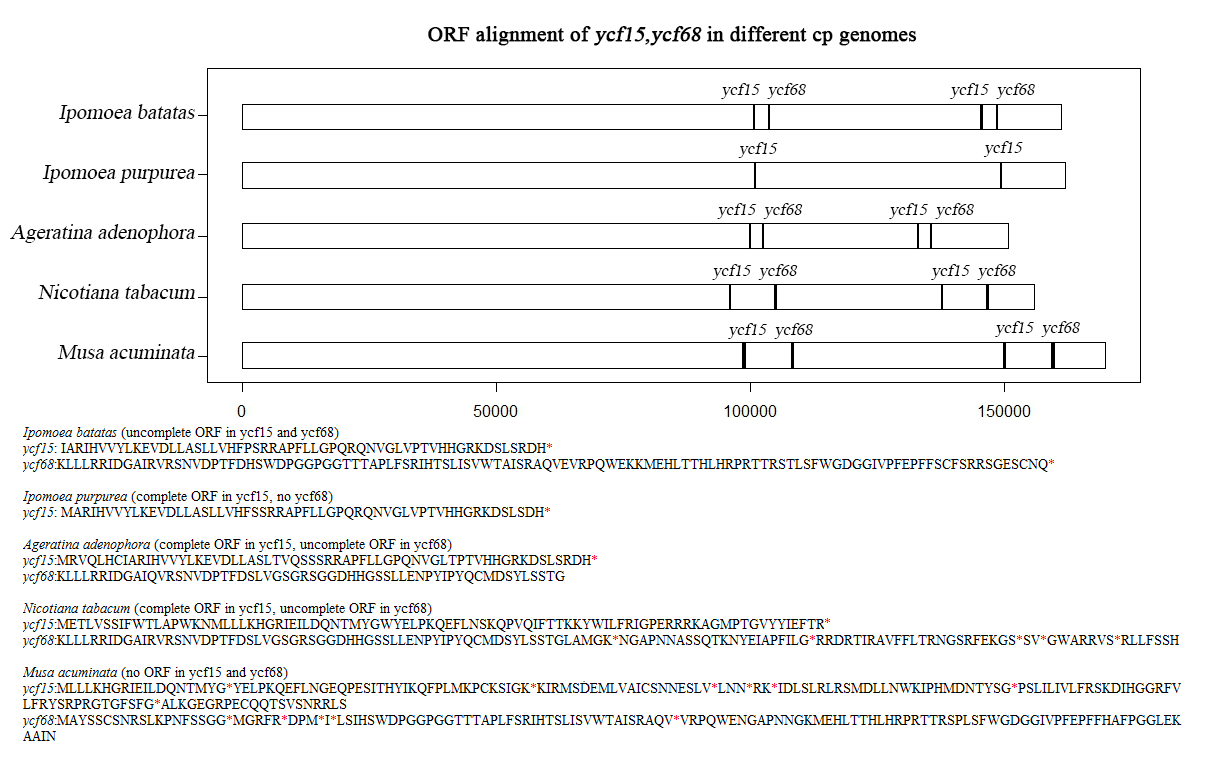

Supplement: S1 Fig — (TIF) [file pone.0124083.s001.tif]
