# Supplementary material for: Analyses of the Complete Genome and Gene Expression of Chloroplast of Sweet Potato [Ipomoea batata]
Source: PLoS One. 2015 Apr 15;10(4):e0124083. doi: 10.1371/journal.pone.0124083 (PMC4398329; doi:10.1371/journal.pone.0124083)
Supplement: S1 Table — (DOC) [file pone.0124083.s002.doc]

| Ib-ch1F | 5’- GTAATAGTGCGAAAAGAAGGCCTG-3’ |
| --- | --- |
| Ib-ch1R | 5’- GATGTAGTGACTGAGGGGGTCG -3’ |
| Ib-ch2F | 5’-TAGATGGTGAAAGTCCAGTAGCC -3’ |
| Ib-ch2R | 5’-CATCGATTCTCCCGAGAGTTCC -3’ |
| Ib-ch3F | 5’-GATTCTCCCGAGAGTTCCGAG -3’ |
| Ib-ch3R | 5’-GTGAAAGTCCAGTAGCCGAAAG -3’ |
| Ib-ch4F | 5’-CATTGCACTGATGTAGTGACTGAG-3’ |
| Ib-ch4R | 5’-GGGCAGTTGACAATTGAATCCG-3’ |
| Ib-ch5F | 5’-GGCAGAATTTCATTGTCAAAGCAAG-3’ |
| Ib-ch5R | 5’-AGACATAGTGGTTGTCTGTCTAACG-3’ |
| Ib-ch6F | 5’-GGTATGGAAACTTACTAAGTGATAAC-3’ |
| Ib-ch6R | 5’-CTTACGAGAATAGAGTATGGTGTTACG-3’ |
| Ib-ch7F | 5’-TTTCCCACTAAGTGATTTATATGAATC-3’ |
| Ib-ch7R | 5’-TATAGATCCAATGTCACATTCCG-3’ |
| Ib-ch8F | 5’-GTAGAAGAACCCAGATTCCAAATG-3’ |
| Ib-ch8R | 5’-GCTGAAGCTGAAGCAGCTAC-3’ |

**TableS1. List of primers used to complete gap regions and RT-PCRs.**

**primers used to complete gap regions:**

**primers used to RT-PCR:**

| Ib-RT1-F | 5’-CAAGGGAAAAGGATTACAGCTTTC-3’ |
| --- | --- |
| Ib-RT1-R | 5’-GATGAGTAAATCTAGGATTTCGTATTCTCG-3’ |
| Ib-RT2-F | 5’-GCTTGCTCGATCTGTGCAATTG-3’ |
| Ib-RT2-R | 5’-GAGGCTGATCTTGAGCGGCC-3’ |
| Ib-RT3-F | 5’-GGTAATACAGAGGATGCAAGCGTTATC-3’ |
| Ib-RT3-R | 5’-CACCTTCCTCCGGCTTATCAC-3’ |
| Ib-RT4-F | 5’-AAGAAACGAAGTGACCTGGACCAC-3’ |
| Ib-RT4-R | 5’-GATTTCTTTC ACCGATTCCATGAAC-3’ |
| Ib-RT5-F | 5’-GATTGCAATATCGGGTAACCCTGTC-3’ |
| Ib-RT5-R | 5’-GTACCGGATTCAGAGGATTAGTGC-3’ |
| Ib-RT6-F | 5’-AGTCCGAGTGGCGGCATACC-3’ |
| Ib-RT6-R | 5’-GACCAGAAGTGCTACTGATAATAATGATC-3’ |
| Ib-RT7-F | 5’-GCTCAGGGGATTAGAGCACGTG-3’ |
| Ib-RT7-R | 5’-GTAGAGCGGTCGGCTGTTAACC-3’ |
| Ib-RT8-F | 5’-CATTATGGGGGAAGTTGATCGTTG-3’ |
| Ib-RT8-R | 5’-GCGAATTCGCTTTCCAATATATCG-3’ |
| Ib-RT9-F | 5’-TGATTAATTTGTATGACCATCGAGGG-3’ |
| Ib-RT9-R | 5’-CTAATCAATTGGACAATGCTTGGG-3’ |
| Ib-RT10-F | 5’-TTGGAACGAGCACAAGCTACTATACAAG-3’ |
| Ib-RT10-R | 5’-GACTGGGCCTATAATCGTGCTTATATAG-3’ |
| Ib-RT11-F | 5’-GTTGGAGATAAGCGGACTCGAAC-3’ |
| Ib-RT11-R | 5’-GGGTCGGAGAAGGGCAATC-3’ |
| Ib-RT12-F | 5’-ATAGACCATCGATCAGTTGATTCGTTC-3’ |
| Ib-RT12-R | 5’-CCATCTCTCCTACATAATTATTATGCC-3’ |
| Ib-RT13-F | 5’-CTAGCGAAGCAAGAAATTGCATTG-3’ |
| Ib-RT13-R | 5’-GCTTGTTTTAGCCGATCTTAGTATTGG-3’ |
| Ib-RT14-F | 5’-GTTATCCGCTCCGCACTTG-3’ |
| Ib-RT14-R | 5’-GGACGAAAGTCGGCCTTAGTGATC-3’ |
| Ib-RT15-F | 5’-CATGTCACGTCGAGGTACTGC-3’ |
| Ib-RT15-R | 5’-GAGTCGATCCACCTACACGTCTTG-3’ |

**primers used to qRT-PCR:**

| Ib-psaA-F | 5’-ATGGGTTACTCCTACAGCACG -3’ |
| --- | --- |
| Ib-psaA -R | 5’-TGGGCACAGGCATCTCAG -3’ |
| Ib-psbI-F | 5’-CCTCTTCCAGGTCCATCACAA -3’ |
| Ib-psbI -R | 5’-TTGCTCGTAGTTCCCGTTTGA -3’ |
| Ib-petB-F | 5’-GATGGTCGGCAAGTATGATGG -3’ |
| Ib-petB-R | 5’-TGGTCCCGAGGTAAGGAATAA -3’ |
| Ib-atpH-F | 5’-GTGCTAATGCCACAACCAATCC -3’ |
| Ib-atpH-R | 5’-GGTCAAGCTGTAGAGGGTATCG -3’ |
| Ib-ndhB-F | 5’-TACAACTCCCATCCCACCTAA -3’ |
| Ib-ndhB-R | 5’-CACTTCTATACGAAATGCTGAT -3’ |
| Ib-accD-F | 5’-AGTAGACGAACTCATACGAAGGGTG -3’ |
| Ib-accD-R | 5’-TATTCATCCATAGGCTCCCAC -3’ |
| Ib-rpl23-F | 5’-TCTGTCCCATTCTTCTACTCT -3’ |
| Ib-rpl23-R | 5’-CAAGAGCAACTAAGATAGGAATA -3’ |
| Ib-rps8-F | 5’-CCTTCTATGTCGCAGGGTTA -3’ |
| Ib-rps8-R | 5’-TGGGTAGGGACACTATTGCTG -3’ |
| Ib-rpoC1-F | 5’-GCATACGACGATTCTTTCACC -3’ |
| Ib-rpoC1-R | 5’-GATGGTGCTGCTACGGTTGAG -3’ |
