# Supplementary material for: Analyses of the Complete Genome and Gene Expression of Chloroplast of Sweet Potato [Ipomoea batata]
Source: PLoS One. 2015 Apr 15;10(4):e0124083. doi: 10.1371/journal.pone.0124083 (PMC4398329; doi:10.1371/journal.pone.0124083)
Supplement: S2 Table — (DOC) [file pone.0124083.s003.doc]

**Table S2. Accessions for taxa used in phylogenetic reconstruction and genome comparison in this study.**

| **Taxon** | **GenBank accession number** |
| --- | --- |
| Ipomoea amnicola | KF242478.1 |
| Ipomoea trifida | KF242496.1 |
| Ipomoea ternifolia | KF242494.1 |
| Ipomoea hederifolia | KF242484.1 |
| Ipomoea cairica | KF242480.1 |
| Ipomoea eriocarpa | KF242483.1 |
| Ipomoea involucrata | KF242485.1 |
| Ipomoea obscura | KF242499.1 |
| Ipomoea diamantinensis | KF242481.1 |
| Cuscuta exaltata | EU189132.1 |
| Nicotiana tabacum | Z00044.2 |
| Solanum tuberosum | DQ231562.1 |
| Solanum lycopersicum | DQ347959.1 |
| Capsicum annuum | JX270811.1 |
| Datura stramonium | JN654342.1 |
| Atropa belladonna | AJ316582.1 |
| Olea europaea | GU931818.1 |
| Coffea Arabica | EF044213.1 |
| Ageratina adenophora | JF826503.1 |
| Daucus carota | DQ898156.1 |
| Populus trichocarpa | EF489041.1 |
| Glycine max | DQ317523.1 |
| Medicago truncatula | AC093544.8 |
| Arabidopsis thaliana | AP000423.1 |
| Gossypium hirsutum | DQ345959.1 |
| Oryza sativa | JN861110.1 |
| Sorghum bicolor | EF115542.1 |
| Zea mays | X86563.2 |
| Calycanthus fertilis | AJ428413.1 |
| Amborella trichopoda | AJ506156.2 |
| Vitis vinifera | DQ424856.1 |
| Saccharum officinarum | AP006714.1 |
